# From Resource Activation to Utilization: A Process‐Outcome Analysis in Telephone‐Based Cognitive‐Behavioral Therapy for Family Caregivers of People With Dementia

**DOI:** 10.1002/jclp.70180

**Published:** 2026-07-15

**Authors:** Nils F. Töpfer, Robin Wester, Gabriele Wilz

**Affiliations:** ^1^ Institute for Clinical Psychology and Psychotherapy, MSH Medical School Hamburg Hamburg Germany; ^2^ Clinical Institute of Psychosomatic Medicine and Psychotherapy Heinrich‐Heine‐University Düsseldorf Düsseldorf Germany; ^3^ Department of Counselling and Clinical Intervention, Institute of Psychology Friedrich‐Schiller‐University Jena Jena Germany

**Keywords:** candidate mechanism of change, cognitive‐behavioral therapy, dementia, family caregivers, process‐outcome analysis, resource activation

## Abstract

**Objective:**

This study aimed to assess whether resource activation (i.e., fostering strengths and healthy aspects of the patient), aggregated across therapy sessions during telephone‐based cognitive‐behavioral therapy (TEL‐CBT), is associated with post‐therapy psychosocial resource utilization (i.e., the extent to which individuals utilize resources to achieve motivational goals) among family caregivers of people with dementia, controlling for baseline resource utilization. Additionally, we analyzed the trajectory of resource activation and investigated predictors of interindividual differences in growth patterns, as well as phase‐specific associations with post‐therapy resource utilization.

**Methods:**

One hundred and twenty family caregivers received 12 sessions of TEL‐CBT. Caregivers and therapists rated resource activation after each session. Caregivers rated utilization of resources related to well‐being, coping with daily hassles, and social support before and after therapy.

**Results:**

Multiple linear regression analyses indicated that mean resource activation aggregated across therapy sessions, as reported by caregivers but not therapists, was significantly associated with post‐therapy resource utilization after adjusting for baseline levels. Using piecewise latent growth curve models, we found that caregiver‐rated resource activation at the beginning of therapy was significantly associated with post‐therapy resource utilization related to well‐being and social support. Pre‐therapy utilization of social support resources was negatively associated with caregiver‐rated resource activation in the final therapy phase, and utilization of well‐being resources before therapy was positively related to therapist‐rated resource activation at the beginning of therapy.

**Conclusion:**

The findings are consistent with the interpretation of resource activation experienced by patients as a candidate mechanism of change. Early resource activation during therapy appears particularly relevant.

## 1 Introduction

Mental health has traditionally been viewed as a one‐dimensional spectrum of psychological suffering (Slade 2010). Accordingly, psychotherapy has long focused predominantly on treating patients based on their specific disorder‐relevant deficits and vulnerabilities. A bi‐dimensional approach recognizes the significance of considering psychosocial strengths alongside problems and suffering. The core principle of *strength‐based methods* (SBM) in psychotherapy is that leveraging patients' strengths is crucial for therapeutic change. SBM aim to strike a balance between enhancing patients' strengths and addressing their deficits and challenges. These methods, also referred to as positive interventions, resilience‐based, or resource‐oriented approaches, involve therapist behaviors that recognize, validate, and encourage the client's strengths, capabilities, and readiness for change in therapy (Flückiger et al. 2023). By addressing both maladaptive and functional aspects of behavior, SBM provide a more comprehensive understanding of mental health. From this perspective, *compensation models* that address patients' deficits and *capitalization models* that build on their strengths are considered complementary rather than contradictory.

Meta‐analyses have demonstrated small to medium effects of SBM on mental health outcomes in clinical samples compared to control conditions (Chakhssi et al. 2018; Munder et al. 2019). Moreover, Flückiger et al. (2023) investigated the potential added contribution of SBM to bona fide psychotherapy, that is, whether SBM enhance the efficacy of established psychotherapies designed to be fully therapeutic. The comparative meta‐analysis found a small but significant effect in favor of strength‐based psychotherapies.

However, *treatment package designs* do not allow for drawing conclusions about specific change mechanisms but only about the treatment as a whole (Flückiger et al. 2017). Consequently, it remains unclear whether treatment effects stem from the specific treatment component assumed to be the working mechanism. To address this, process‐outcome studies assessing hypothesized working mechanisms throughout therapy are needed.

### 1.1 Resource Activation as a General Strength‐Based Change Mechanism of Psychotherapy

One of the most important working mechanisms of interest in SBM is *resource activation*. Based on a review of the psychotherapy literature, Grawe (1997) identified resource activation as one of four general change mechanisms of psychotherapy, that is, a mechanism that all therapies have in common. He argued that activating resources is the key to psychotherapeutic change, considering it “the alpha and omega of effective therapy” (p. 6).

According to Grawe's (2004) consistency theory, people develop psychosocial resources as means for achieving their motivational goals (as individual operationalizations of basic psychological needs). The mechanism through which patients' resources, previously not consciously accessible, are activated is called resource activation. Resource activation is assumed to lead to higher *resource utilization*, that is, the degree to which individuals actually utilize and apply their resources for reaching a motivational goal. Resource activation is thought to initiate an upward spiral: When patients are enabled to experience themselves as competent in therapy, their mood improves, which facilitates the accessibility of positive memories. As a result of an increased sense of mastery, positive expectancy is induced, which leads to need‐fulfilling experiences through a self‐fulfilling prophecy. This sense of achievement enhances the patient's confidence in the therapy's effectiveness, strengthens the working alliance, and increases their engagement in therapy. Thereby, the conditions for all other therapeutic components to become active and effective are enhanced. By facilitating problem‐solving attempts, resource activation can help alter disorder patterns and reduce symptoms.

Resource activation measured in the therapy process has been shown to be associated with symptom reduction and global therapy outcome (Call et al. 2018; Mander et al. 2013). Global resource activation and the quality of resource activation were significantly related to higher levels of attaining individualized therapy goals by the 10th therapy session (Flückiger et al. 2009). However, to our knowledge, no process‐outcome study has so far analyzed the association of resource activation measured over the therapy process with resource utilization after therapy. According to consistency theory, resource utilization is the most direct indicator of successful resource activation. A higher degree of resource utilization is associated with better mental health, improved well‐being, lower psychopathological symptomatology, reduced motivational incongruence, and enhanced interpersonal skills (Trösken 2002). Resource utilization predicted 1‐year post‐therapy outcome in inpatients of a psychosomatic rehabilitation clinic (Deubner‐Böhme et al. 2011), as well as alcohol‐dependent inpatients (Deppe‐Schmitz et al. 2009). In addition, improvements in resource utilization during psychosomatic treatment were associated with all measures of therapy success at the end of treatment (Groß et al. 2014).

Given that psychotherapy unfolds across multiple sessions and phases, examining the therapy process offers valuable opportunities to explore the course and timing of resource activation. Such insights could inform the development of therapeutic heuristics. To date, no study has investigated the trajectory of resource activation throughout therapy while accounting for its phased structure.

### 1.2 Resource Activation in Interventions for Family Caregivers of People With Dementia

A population that might particularly benefit from resource activation is family caregivers of people with dementia. They are confronted with challenges and changes that often have a negative impact on their health and well‐being. Recent meta‐analyses report a pooled prevalence of depression at 31.2%, anxiety at 32.1%, and burden at 49.3% (Collins and Kishita 2020; Kaddour and Kishita 2020). Psychotherapeutic support may therefore be reasonable or even necessary for a substantial proportion of family caregivers of people with dementia.

The importance of resources for family caregivers becomes particularly evident in dealing with stressors: Caregivers differ in how burdensome they find caregiving. This variation is not explained by *objective* stressors but rather by the *subjective* appraisal of the caregiving situation, which in turn depends on the perceived availability of coping *resources* (Nolan et al. 1996). Activating coping‐related resources is critical given the ever‐changing and progressively demanding nature of caregiving, which requires continuous adaptation to new challenges (Gottlieb and Wolfe 2002; Kneebone and Martin 2003). Because many caregivers experience social isolation and a lack of appropriate emotional support, activating resources related to social connectedness is particularly relevant (Stoltz et al. 2004).

While interventions often focus primarily on mitigating strain and emotional burden, it is equally important to strengthen caregivers' capacity to experience pleasure, engagement, and personal fulfillment. Family caregivers of people with dementia frequently neglect or give up self‐care, leisure, and other restorative activities due to their caregiving responsibilities, which has been linked to poorer physical and mental health outcomes (Ory et al. 1999; Schüz et al. 2015).

Taken together, these findings highlight the relevance of resource activation as a central therapeutic principle in interventions for family caregivers of people with dementia – one that aims to reduce both negative experiences and enhance positive ones.

Research on strengths‐based interventions aimed at fostering resources in family caregivers of people with dementia remains limited, with even fewer studies examining strength‐based change mechanisms (Töpfer and Wilz 2018, 2021). Among the existing strength‐based programs, coping‐, wellness‐, or resourcefulness‐focused interventions have demonstrated promising effects on caregivers' well‐being and coping abilities, yet most rely on cross‐sectional or pilot designs and, while theoretically grounded in related frameworks, have not been linked to resource activation as a general change mechanism of psychotherapy (Töpfer and Wilz 2018). Existing research on mechanisms of change has primarily focused on post‐intervention mediators, leaving open questions about the mechanisms at work during the intervention. For example, previous mediation studies have predominantly examined distress‐reducing mechanisms (e.g., reductions in dysfunctional thoughts or improvements related to coping and stress), while only few studies have examined gain‐enhancing mediators – such as self‐efficacy and caregiving gains (Cheng et al. 2016) or leisure activities (Losada et al. 2011). Together, these findings highlight the need for process‐based investigations of resource‐activating mechanisms within strength‐based interventions to assess these mechanisms during the intervention period.

Tele.TAnDem, a telephone‐based cognitive‐behavioral therapy (CBT) intervention for family caregivers, incorporates resource activation as a superordinate intervention heuristic (Töpfer and Wilz 2018). It significantly increased caregivers' post‐therapy utilization of well‐being and coping resources compared to a control group (Töpfer and Wilz 2018), with this increase mediating improvements in caregivers' quality of life at post‐test and 6‐month follow‐up (Töpfer and Wilz 2021). Two process‐outcome analyses of Tele.TAnDem examined associations between process variables, including resource activation, assessed after each session by caregivers and therapists, and treatment outcomes. Resource activation in internet‐based Tele.TAnDem for family caregivers of people with dementia did not predict treatment outcomes (Theurer et al. 2021). Only therapist‐rated, but not caregiver‐rated, resource activation over the course of telephone‐based Tele.TAnDem significantly reduced caregiver burden in distressed family caregivers (not limited to caregivers of people with dementia) (Wrede et al. 2023). Thus, further process‐outcome analyses of resource activation are necessary. In particular, no prior study on telephone‐based CBT has examined whether resource activation measured during therapy is associated with post‐therapy psychosocial resource utilization, a proximal indicator of successful resource activation. Moreover, previous research has relied on mean levels of resource activation across sessions; consequently, the trajectory and timing of resource activation have not yet been examined in relation to post‐treatment resource utilization. The present study addresses these gaps by examining both mean levels and phase‐specific growth patterns of resource activation across 12 sessions of telephone‐based CBT and their associations with post‐therapy psychosocial resource utilization.

Regarding the trajectory of resource activation across therapy, the present study modeled nonlinear change across three phases, reflecting both the structure of Tele.TAnDem and the empirically derived phase model of psychotherapy outcome proposed by Howard et al. (1993). This model distinguishes three successive stages of change: enhanced subjective well‐being (remoralization), followed by symptom reduction (remediation), and finally improvements in broader functioning domains such as relationships, self‐care, or life enjoyment (rehabilitation). Previous research has supported the model's temporal structure, showing that well‐being tends to change fastest, followed by symptoms and then interpersonal functioning (Sembill et al. 2019). Partial support has also been found in outpatient settings (Callahan et al. 2006) and in studies on clinically significant deterioration, where demediation reliably preceded dehabilitation, while demoralization did not consistently occur first (Swift et al. 2010).

The significance of hope during the remoralization phase – as a predictor of subsequent symptom reduction – has been supported by several findings (Abel et al. 2016; Hayes et al. 2007; Kuyken 2004). Resource activation does not represent an unspecific curative factor that automatically operates during the remoralization phase (Flückiger et al. 2009). Rather, it initiates the previously mentioned upward spiral in which need‐satisfying experiences enhance well‐being and, through increased receptivity and engagement in therapy, contribute to subsequent symptom reduction, with need‐fulfilling experiences gradually overriding maladaptive patterns and establishing more adaptive ones. The potential influence of resource activation thus extends across all three phases of Howard et al. (1993) model.

### 1.3 Research Objective

The present investigation aimed to assess whether resource activation, measured from the caregiver's and the therapist's perspective using post‐session report forms, is associated with post‐therapy psychosocial resource utilization of family caregivers of people with dementia as a proximal indicator of successful resource activation following telephone‐based CBT. We hypothesized that higher levels of resource activation would be associated with greater psychosocial resource utilization at post‐treatment.

Additionally, we examined the trajectory of resource activation across the 12 therapy sessions and investigated predictors of interindividual differences in growth patterns for each phase, as well as phase‐specific associations of resource activation with post‐therapy psychosocial resource utilization. Given that previous evidence on the temporal course of resource activation stems mainly from in‐session or phase‐comparative studies (e.g., Smith and Grawe 2005; Gassmann and Grawe 2006) and not from longitudinal analyses across an entire treatment – particularly not in telephone‐based CBT or other interventions for family caregivers of people with dementia – these analyses were exploratory in nature.

## 2 Materials and Methods

The current study is based on data from a randomized controlled trial evaluating the effectiveness of Tele.TAnDem for family caregivers of people with dementia, implemented in established care provision structures, relative to usual care (Soellner et al. 2015).

### 2.1 Procedure

Family caregivers of people with dementia were recruited nationwide. Inclusion criteria required participants to be the primary caregiver of a person diagnosed with at least mild dementia according to the Global Deterioration Scale (Reisberg et al. 1982). Exclusion criteria included ongoing psychotherapy, severe physical or psychiatric disorders, or plans to institutionalize the care recipient within 6 months. After baseline assessment (T0), caregivers were randomized to the Tele.TAnDem intervention group (TEL‐IG) or usual care (control group). Participants completed further assessments 6 months after baseline, following completion of all therapy sessions (T1), 12 months after baseline (T2), and 3 years after baseline (T3). For the present investigation, only data from TEL‐IG at baseline and psychosocial resource utilization at post‐treatment were analyzed. The trial was approved by the Ethics Committee of Friedrich‐Schiller‐University Jena, and all participants provided written informed consent.

### 2.2 Intervention

Participants in TEL‐IG received 12 therapy sessions, each lasting 50 min, delivered via telephone over 6 months. Sessions 1–4 were conducted weekly, sessions 5–10 biweekly, and sessions 11–12 monthly.

Fifteen qualified clinical psychologists (93.34% female, age: *M* = 31.47, *SD* = 4.17) conducted the therapies. They had either completed (*n* = 4, 26.7%) or were in an advanced stage of post‐graduate CBT training (*n* = 11, 73.3%), with an average of 4.27 years of clinical experience (*SD* = 3.41). All therapists completed an 8‐h preintervention workshop on the Tele.TAnDem manual and received regular supervision. Supervision was conducted primarily in full‐day group sessions and supplemented, when needed, by individual face‐to‐face or telephone supervision. Therapists rated the supervision as helpful to very helpful (*M* = 3.50/4, *SD* = 0.65), and the available supervision formats were used consistently throughout the intervention period. Because the specific frequency and format varied across therapists depending on clinical needs, no fixed or standardized supervision schedule was implemented.

The manual includes 10 modules that can be used and combined based on the individual needs and goals of each caregiver (Wilz 2024): (a) basic elements (e.g., establishing a therapeutic alliance); (b) problem analysis (identification of main problems using functional behavioral analysis and therapy goals); (c) psychoeducation (caregiving and disease‐related information); (d) strengthening problem solving abilities; (e) changing dysfunctional cognitions; (f) increasing the use of informal and professional support; (g) coping with change, grief, and loss; (h) self‐care and value‐based activities (e.g., planning health‐promoting activities); (i) stress‐management and emotion regulation (e.g., anger and tension management); and (j) evaluation and future planning (e.g., reviewing progress and setting future goals).

Resource activation was integrated as a superordinate intervention heuristic through resource‐focused questions, a resource‐oriented therapeutic stance, and a complementary therapeutic relationship tailored to each caregiver's motivational resources (Töpfer and Wilz 2018). Therapists were encouraged to acknowledge caregivers as “care experts” and validate their caregiving efforts.

Expert ratings of audiotaped sessions indicated therapists' adherence to the manual and competence, rated as high to very high (Hillebrand et al. 2021; Wilz et al. 2018).

### 2.3 Assessments

#### 2.3.1 Psychosocial Resource Utilization

The Psychosocial Resource Utilization Questionnaire (PRUQ; Töpfer and Wilz 2018) measured how frequently caregivers utilized psychosocial resources to achieve motivational goals in three areas over the past 4 weeks, rated on a 5‐point scale (1 = *never* and 5 = *very often*). *Utilization of resources related to well‐being* was assessed using 9 items (e.g., “… I spent time with friends,” “… I did something for my health and physical fitness”; *α* = 0.85). *Utilization of resources related to coping with daily hassles* was measured with 20 items (e.g., “… to take a step back and figure things out,” “… to accept things as they are”; *α* = 0.76). *Utilization of resources related to social support* was evaluated using 7 items (e.g., “… treated me thoughtfully,” “… encouraged me”; *α* = 0.87).

#### 2.3.2 Resource Activation

Consistent with its conceptualization as a session‐bound process, resource activation was assessed after each therapy session by therapists and caregivers, using the Bern Post Session Report Form 2000, which measures central aspects of the therapy process (Flückiger et al. 2010). For the present study, the *resource activation* subscale was used. From the therapist's perspective, resource activation was rated on a 5‐point scale (0 = *not at all* and 4 = *absolutely right*) using 3 items (e.g., “Today, I tried to capitalize on the psychosocial resources of my patient in a targeted way”; *α* = 0.87). From the caregiver's perspective, resource activation was assessed on a 7‐point scale (−3 = *not at all*, +3 = *yes, exactly*) using 3 items (e.g., “In today's session, my therapist enabled me to feel where my strengths lie”; *α* = 0.81). For each session, the correlation between therapist‐ and caregiver‐rated resource activation was small and non‐significant (range: *r* = −0.10 to 0.16).

### 2.4 Data Analysis

We applied a two‐step approach to analyze associations between resource activation and psychosocial resource utilization. First, we examined whether mean resource activation over the 12 therapy sessions (analyzed separately from the therapist's and caregiver's perspective) was associated with post‐therapy utilization of resources (analyzed separately for resources related to well‐being, coping with daily hassles, and social support). These analyses constituted the hypothesis‐driven tests of the study. Mean resource activation represents an aggregated treatment‐period process variable. Using multiple regression analysis, we assessed whether mean resource activation explained additional variance in psychosocial resource utilization beyond baseline levels. Accordingly, regression coefficients for mean resource activation reflect associations with post‐treatment psychosocial resource utilization adjusted for baseline levels. Additionally, we tested the interaction between baseline resource utilization and mean resource activation to determine whether the association between resource activation and post‐therapy resource utilization depends on baseline resource utilization. To clarify the structure of the regression analyses, we estimated three consecutive models for each resource domain (well‐being, coping with daily hassles, and social support). Model 1 included only baseline resource utilization as a predictor of post‐treatment resource utilization. Model 2 added intraindividual mean resource activation across the 12 sessions, and Model 3 included the interaction between baseline resource utilization and mean resource activation. Nested model comparisons (ANOVA) were used to determine whether each successive model accounted for additional variance beyond the preceding model.

Second, we used latent growth curve modeling (LGM) to investigate the trajectory of resource activation. These analyses were exploratory in nature, as they were not based on specific a priori hypotheses. We specified a piecewise LGM with three slopes to model nonlinear change, as Tele.TAnDem was conducted in three therapy phases with varying session intervals (sessions 1–4 weekly, sessions 5–10 biweekly, and sessions 11–12 monthly). This approach aligns with Howard et al. (1993) phase model of psychotherapy outcome, which distinguishes three phases: remoralization (enhanced well‐being), remediation (symptom resolution), rehabilitation (maintenance of gains and improved functioning). Including three slopes ($\beta_{1},\beta_{2},\beta_{3}$) – along with one intercept (α) – allowed us to model changes in the trajectory of resource activation, with each slope representing the rate of change in the corresponding therapy phase. This model allowed us to examine baseline resource utilization as predictors of interindividual differences in growth parameters for each therapy phase and to analyze phase‐specific associations of resource activation with post‐therapy psychosocial resource utilization. In the latter analyses, both baseline resource utilization and the latent growth parameters (intercept and three slopes) were included as predictors. Accordingly, associations between growth parameters and post‐treatment psychosocial resource utilization reflect relationships with outcomes adjusted for baseline levels. These growth parameters represent change processes that temporally precede the post‐treatment outcome but are not adjusted for earlier phase‐specific change, because they are estimated simultaneously. The intercept and slopes were freely estimated and allowed to covary. All predictors and outcomes were estimated within a single piecewise LGM for each perspective (caregiver and therapist).

To analyze the trajectory of resource activation throughout the entire therapy course, we conducted a completer analysis, limiting results to caregivers who successfully completed therapy.

All analyses were performed using the R programming language (Version 3.6) (R Core Team 2019) and the R package lavaan (Version 0.6) for LGM (Rosseel 2012). All variables included in the analyses were screened for univariate skewness and kurtosis and visually inspected. Because these checks indicated slight deviations from normality for some variables, all LGMs were estimated using the robust version of full information maximum likelihood (FIML) in lavaan.

Missing data rates were low across the variables used in the regression and LGMs (overall missingness: 2%). Because no systematic patterns of missingness were observed (e.g., across therapy phases), the missing‐at‐random assumption appears plausible. Accordingly, all models were estimated using robust FIML.

Error variances of the repeated measurements of resource activation were allowed to vary across time points. The full analysis syntax for the regression models and the piecewise LGMs is provided in the online Supplemental Material.

## 3 Results

### 3.1 Sample Characteristics

Characteristics of caregivers and care recipients are shown in Table 1.

**Table 1:** Demographic characteristics of caregivers and care recipients. Percentage or means with standard deviations and ranges in parentheses.

Variable	Caregiver	Care recipient
Age (years)	63.3 (11.4; 23–85)	79.1 (9.01; 55–99)
Female	83%	51%
Living with care recipient	80%	—
Duration of caregiving (years)	4.0 (3.4; 0–19)	—
Relationship to care recipient		
Spouse/partner	57%	—
Daughter/son	42%	—
Other	1%	—
Type of dementia		
Alzheimer's disease	—	45.0%
Vascular dementia	—	14.2%
Frontotemporal dementia	—	3.3%
Other	—	37.5%
Severity of dementia^a^		
Mild	—	0.8%
Moderate	—	2.5%
Moderately severe	—	35.3%
Severe	—	36.1%
Very severe	—	25.2%

^a^ Severity of dementia was measured using the Global Deterioration Scale (Reisberg et al. 1982).

### 3.2 Drop‐Outs

Of the 139 participants randomized to TEL‐IG, 120 (86.3%) completed the intervention. Reasons for discontinuing therapy among the 19 non‐completers included lack of time (*n* = 6), death of the care recipient (*n* = 2), lack of energy (*n* = 1), perceived intervention burden (*n* = 1), institutionalization of the care recipient (*n* = 1), lack of supervision for care recipient during sessions (*n* = 1), and personal illness (*n* = 1).

There were no significant demographic differences between completers and non‐completers, except that a significantly higher proportion of non‐completers (94%) lived with the care recipient compared to completers (80%).

### 3.3 Sample Sizes Across Analyses

Differences in sample size across analyses are due to variation in data availability. Two participants did not provide post‐treatment data and were therefore excluded from the regression analyses (*N* = 118). One of these participants also did not provide process data and was therefore excluded from the unconditional LGM (*N* = 119). The conditional LGM included all participants (*N* = 120), with missing data handled using full information maximum likelihood (FIML).

### 3.4 Associations of Average Resource Activation With Post‐Therapy Resource Utilization

The following section presents the results of the hypothesis‐driven regression analyses examining associations between resource activation averaged over the 12 therapy sessions and post‐therapy psychosocial resource utilization (Tables 2 and 3; *N* = 118). Mean resource activation from the caregiver's perspective was significantly associated with post‐therapy resource utilization after controlling for baseline levels. Table 2 presents unstandardized and standardized regression coefficients, as well as *R* ^2^ values for each area of resource utilization. Mean resource activation explained an additional 4% of the variance in the utilization of resources related to well‐being and coping with daily hassles, and 5% in the utilization of resources related to social support post‐therapy, beyond baseline resource utilization, resulting in significant but small associations. Caregivers who reported higher levels of resource activation throughout therapy utilized resources related to well‐being, coping with daily hassles, and social support significantly more frequently after therapy, above and beyond baseline levels.

**Table 2:** Results of the regression analyses predicting post‐treatment (T1) resource utilization from baseline (T0) resource utilization and mean caregiver‐rated resource activation across 12 sessions (*N* = 118).

Coefficient	*R* ^2^	*B* (*SE*)	*β*
Social support T1	0.28		
Intercept		0.79 (0.33)*	
Resource utilization T0		0.54 (0.09)***	0.46
Mean resource activation		0.35 (0.12)**	0.23
Well‐being T1	0.32		
Intercept		0.95 (0.29)**	
Resource utilization T0		0.58 (0.08)***	0.55
Mean resource activation		0.25 (0.10)*	0.19
Coping with daily hassles T1	0.35		
Intercept		–0.67 (0.84)	
Resource utilization T0		1.25 (0.30)***	1.18
Mean resource activation		1.12 (0.43)*	1.27
Resource utilization T0 × mean resource activation		–0.36 (0.15)*	–1.41

*Note:* Three regression model sequences (one per resource area) were estimated: Model 1 = baseline only; Model 2 = baseline + mean resource activation; Model 3 = baseline + mean resource activation + interaction (resource utilization T0 × mean resource activation). Only the final models, including mean resource activation are displayed; the interaction term was included only for coping with daily hassles, where it was significant.

* *p* < 0.05

** *p* < 0.01

*** *p* < 0.001.

**Table 3:** Results of the regression analyses predicting post‐treatment (T1) resource utilization from baseline (T0) resource utilization and mean therapist‐rated resource activation across 12 sessions (*N* = 118).

Coefficient	*R* ^2^	*B*(*SE*)	*β*
Social support	0.22		
Intercept		1.28 (0.38)**	
Resource utilization T0		0.56 (0.10)***	0.47
Mean resource activation		0.05 (0.11)	0.04
Well‐being	0.30		
Intercept		1.15 (0.30)***	
Resource utilization T0		0.54 (0.08)***	0.51
Mean resource activation		0.13 (0.09)	0.12
Coping with daily hassles	0.31		
Intercept		1.28 (0.27)***	
Resource utilization T0		0.58 (0.08)***	0.55
Mean resource activation		0.05 (0.06)	0.06

*Note:* Three regression model sequences (one per resource area) were estimated: Model 1 = baseline only; Model 2 = baseline + mean resource activation; Model 3 = baseline + mean resource activation + interaction (resource utilization T0 × mean resource activation). Only the final models, including mean resource activation (Model 2) are displayed, as the interaction term was non‐significant in all domains.

* *p* < 0.05

** *p* < 0.01

*** *p* < 0.001.

In contrast, mean resource activation from the therapist's perspective did not show a significant association with caregiver's post‐therapy utilization of resources related to social support, well‐being, or coping with daily hassles (see Table 3).

A significant interaction between baseline resource utilization and mean resource activation from the caregiver's perspective was found for coping with daily hassles. We investigated this interaction by examining the Johnson–Neyman plot using the R package interactions (Long 2019, see Figure S1 in the Supporting Information). The association between resource activation and post‐therapy utilization of coping resources was stronger for caregivers with lower baseline utilization of coping resources. The association was only significant for baseline utilization of coping resources < 2.75, indicating a significant *positive* association between resource activation and post‐therapy utilization of coping resources for baseline values < 2.75, but *not* a significant *negative* association for baseline values > 2.75. The assumptions of linearity, homoscedasticity, and normal distribution of residuals were not violated.

### 3.5 Trajectory of Resource Activation

**Figure 1:**
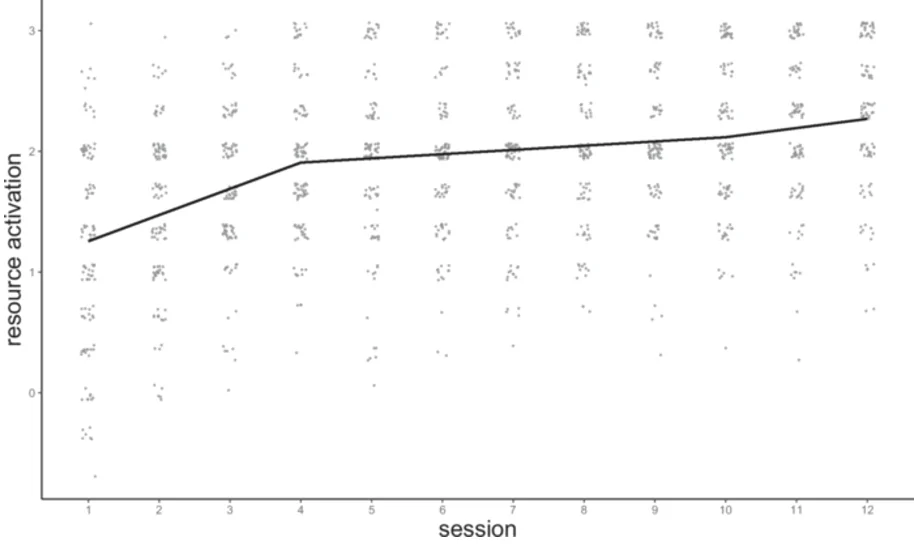
Resource activation from the caregiver's perspective: Predicted average growth trajectory and individual scores.

**Figure 2:**
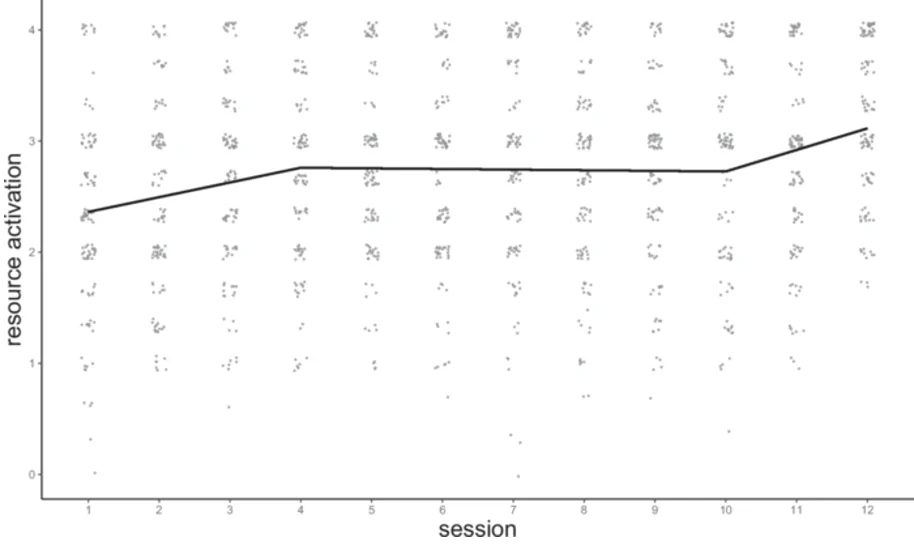
Resource activation from the therapist's perspective: Predicted average growth trajectory and individual scores.

The following section presents the results of the exploratory LGM analyses (Tables 4 and 5, Figures 1 and 2). Participants contributed data across up to 12 therapy sessions, with missing data handled using FIML. We began by estimating an unconditional LGM (caregiver‐rated: *N* = 119; therapist‐rated: *N* = 120) to assess whether the repeated measurements of resource activation conformed to our hypothesized latent curve structure. The model showed a good fit for resource activation from both the caregiver's perspective (*χ* ^2^(64) = 84.79; RMSEA = 0.05, CFI = 0.98, TLI = 0.98) and the therapist's perspective (*χ* ^2^(64) = 75.15; RMSEA = 0.04, CFI = 0.98, TLI = 0.98). Significant variance in growth parameters of resource activation was found for all parameters estimated from the caregiver's perspective and for the first session and middle therapy phase from the therapist's perspective, indicating individual deviations from the average change pattern.

**Table 4:** Means and variances of growth parameters (intercept *α* and slopes *β*_1_–*β*_3_) and regression coefficients from the piecewise latent growth model of resource activation from the caregiver's perspective (conditional model; *N* = 120).

	*α*	*β*_1_	*β*_2_	*β*_3_
Mean	**1.26** ***	**0.22** ***	**0.04** ***	**0.08** **
Variance	**0.384** ***	**0.020** *	**0.004** ***	**0.035** **
Coping T0 →	0.25	–0.01	0.01	0.06
Social support T0 →	–0.08	0.04	0.01	**–0.08** *
Well‐being T0 →	0.01	–0.04	–0.02†	0.07
→ Coping T1	0.18†	0.50	–0.29	0.49
→ Social support T1	**0.40** *	1.01	1.34	0.33
→ Well‐being T1	**0.31** **	–0.04	1.11	0.38

*Note:* All coefficients are taken from a single piecewise latent growth curve model estimated for the caregiver's perspective. Arrows indicate the direction of effects; for example, the row “coping T0 →” contains unstandardized coefficients for regressions of growth parameters on baseline (T0) utilization of coping‐related resources, whereas the row “→ coping T1” contains coefficients for regressions of post‐treatment (T1) utilization of coping‐related resources on baseline resource utilization (coefficients not displayed) and the growth parameters. Significant coefficients are presented in bold.

^†^ *p* < 0.10.

* *p* < 0.05

** *p* < 0.01

*** *p* < 0.001.

**Table 5:** Means and variances of growth parameters (intercept *α* and slopes *β*_1_–*β*_3_) and regression coefficients from the piecewise latent growth model of resource activation from the therapist's perspective (conditional model; *N* = 120).

	*α*	*β*_1_	*β*_2_	*β*_3_
Mean	**2.36** ***	**0.13** ***	–0.01	**0.19** ***
Variance	**0.296** ***	0.004	**0.007** **	0.058
Coping T0 →	0.07	–0.01	0.01	–0.01
Social support T0 →	–0.04	0.01	–0.03	0.03
Well‐being T0 →	**0.25** *	–0.02	0.01	–0.07
→ Coping T1	0.05	0.94	1.24	0.46
→ Social support T1	0.25	–2.84	0.13	0.47
→ Well‐being T1	0.004	5.68	2.54	0.81

*Note:* All coefficients are taken from a single piecewise latent growth curve model estimated for the therapist's perspective. Arrows indicate the direction of effects; for example, the row “coping T0 →” contains unstandardized coefficients for regressions of growth parameters on baseline (T0) utilization of coping‐related resources, whereas the row “→ coping T1” contains coefficients for regressions of post‐treatment (T1) utilization of coping‐related resources on baseline resource utilization (coefficients not displayed) and the growth parameters. Significant coefficients are presented in bold. ^†^ *p* < 0.10.

* *p* < 0.05

** *p* < 0.01

*** *p* < 0.001.

We then extended the unconditional model to a conditional model (caregiver‐rated: *N* = 120; therapist‐rated: *N* = 120) by including baseline resource utilization as predictors of growth parameters and post‐therapy resource utilization as dependent variables. This model was used to examine whether baseline resource utilization was associated with individual differences in phase‐specific growth parameters for the three therapy phases (sessions 1–4, 5–10, and 11–12), and whether these growth parameters were associated with post‐therapy resource utilization. The model fit was moderate to good for the caregiver's perspective (*χ* ^2^(118) = 186.02, RMSEA = 0.07, CFI = 0.95, TLI = 0.94) and the therapist's perspective (*χ* ^2^(118) = 148.53; RMSEA = 0.05, CFI = 0.96, TLI = 0.95). Table 4 shows the means and variances of growth parameters from the caregiver's perspective and Table 5 from the therapist's perspective. On average, resource activation estimated from the caregiver's perspective increased significantly within each therapy phase, with the largest increase occurring in the first phase, leveling off in the middle phase, and rising again at the end of therapy (see Figure 1). Resource activation estimated from the therapist's perspective increased significantly in all therapy phases except the middle phase (see Figure 2).

Baseline utilization of social support resources was significantly negatively associated with the slope of caregiver‐rated resource activation in the last therapy phase, $\beta_{3}$ (*B* = − 0.08), indicating that lower baseline utilization was associated with a greater increase in resource activation toward the end of therapy. Resource activation in the first session (intercept α) from the caregiver's perspective was significantly associated with post‐therapy utilization of resources related to social support (*B* = 0.40) and well‐being (*B* = 0.31), indicating that higher initial resource activation was related to greater post‐therapy resource utilization.

Baseline utilization of well‐being resources was significantly positively associated with therapist‐rated resource activation in the first session (*B* = 0.25), indicating that caregivers who reported higher utilization of well‐being resources before therapy received higher levels of therapist‐rated resource activation in the first session. No other coefficients for regressions of growth parameters on baseline resource utilization or regressions of post‐therapy resource utilization on growth parameters of resource activation from the therapist's perspective were significant.

## 4 Discussion

Building on evidence that SBM enhance psychotherapy efficacy (Flückiger et al. 2023), the present study investigated resource activation (i.e., focusing on patients' strengths and healthy aspects) as a theoretically proposed candidate mechanism of SBM. First, we examined whether resource activation from the caregiver's and therapist's perspective, averaged over the 12 sessions of telephone‐based CBT for family caregivers of people with dementia, was associated with post‐therapy resource utilization. According to Grawe's (2004) consistency theory, resource utilization – the extent to which patients apply available resources to achieve motivational goals – represents a proximal indicator of successful resource activation. In this study, resource utilization was assessed post‐therapy in three areas identified by an expert task force as particularly relevant for family caregivers of people with dementia (Töpfer and Wilz 2018): well‐being, coping with daily hassles, and social support. The results showed that mean resource activation from the caregiver's perspective was significantly associated with the utilization of resources in all three areas post‐therapy. Thus, caregivers who reported higher levels of resource activation during therapy also reported higher utilization of well‐being, coping, and social support resources after therapy. These findings provide the first evidence that resource activation during therapy was associated with higher post‐therapy resource utilization, extending previous research linking resource activation to symptom reduction, global therapy outcome (Call et al. 2018; Mander et al. 2013), and individualized therapy goal attainment (Flückiger et al. 2009).

For utilization of resources related to coping with daily hassles, the analysis revealed a significant interaction between baseline resource utilization and mean resource activation, in addition to the overall association between resource activation during therapy and post‐therapy utilization. This indicates that resource activation showed a stronger association with post‐therapy utilization of coping resources for caregivers who rarely utilized such resources before therapy (values < 2.75 on the 5‐point scale from 1 = never to 5 = very often). For caregivers who already utilized coping resources frequently before therapy, resource activation during therapy was not significantly associated with post‐therapy utilization of coping resources. Caregivers who enter treatment with fewer existing coping resources – and who seem to benefit more strongly from resource activation – may partly represent individuals with limited prior access to supportive mental health services.

These findings highlight the dual role of resource activation in relation to both reductions in negative experiences (e.g., managing stress) and the promotion of positive experiences (e.g., well‐being). While the former appears to have a ceiling, the latter does not seem similarly constrained. By demonstrating that Tele.TAnDem supports highly distressed family caregivers of people with dementia by promoting both coping, well‐being, and social support, this study addresses a gap in caregiver intervention research, which has often prioritized distress reduction while rarely investigating candidate mechanisms for enhancing positive outcomes (Töpfer and Wilz 2021).

Surprisingly, mean resource activation from the therapist's perspective did not show a significant association with post‐therapy psychosocial resource utilization. This finding aligns with the literature on SBM, which emphasizes a client‐centered perspective, suggesting that only those capabilities perceived and positively evaluated by individuals can effectively function as resources. Accordingly, caregiver‐experienced resource activation may be more decisive than therapists' assessments. Consistent with this interpretation, previous studies have shown that patient ratings of general change mechanisms, including resource activation, are better predictors of therapy outcomes than therapist ratings (Call et al. 2018; Mander et al. 2013).

The second objective of this study was to examine the trajectory of resource activation across the 12 therapy sessions and to investigate predictors of interindividual differences in growth patterns for each therapy phase (sessions 1–4, 5–10, and 11–12), as well as phase‐specific associations of resource activation with post‐therapy psychosocial resource utilization. Latent growth curve modeling showed that resource activation from the caregiver's perspective significantly increased in all three therapy phases and in all but the middle phase from the therapist's perspective. From the patient's perspective, the increase was largest in the first phase, leveled off in the second, and rose again in the last phase. This trajectory corresponds with findings on resource activation in successful therapy sessions, where patients experience strong activation at the beginning, a decrease in the middle, and another increase toward the end of the session (Gassmann and Grawe 2006). It also resembles the logarithmic change observed in the global common factor “coping,” which encompasses various general change mechanisms, including resource activation, mastery experiences, self‐efficacy expectations, and positive outcome expectations (Meier et al. 2023).

The analysis of predictors of interindividual differences in growth patterns of resource activation revealed that baseline utilization of social support resources was significantly negatively associated with caregiver‐rated resource activation in the last therapy phase: caregivers who utilized less social support before therapy showed a stronger increase in resource activation toward the end of treatment. As the final phase emphasizes transferring therapeutic gains into daily life, caregivers with limited prior use of social support resources may have relied more strongly on their therapists' assistance, prompting therapists to foster caregivers' self‐efficacy in utilizing alternative support resources beyond therapy.

From the therapist's perspective, caregivers' baseline utilization of well‐being resources was associated with higher therapist‐rated resource activation at the beginning of therapy. One plausible explanation is that caregivers who already drew on well‐being resources before treatment may have been more inclined to reference them in early sessions, giving therapists greater opportunity to recognize and further foster them.

Caregiver‐rated resource activation at the beginning of therapy was significantly associated with post‐therapy utilization of well‐being and social support resources. This finding aligns with Howard et al. (1993) remoralization phase, which emphasizes mobilizing hope and existing coping resources to enhance early subjective well‐being. Such early activation may facilitate subsequent therapeutic work, consistent with findings by Smith and Grawe (2005) showing that early resource activation helps patients perceive that they possess the necessary resources to cope with challenging emotions triggered by problem actuation. The leveling‐off of resource activation in the middle phase may reflect a shift toward more problem‐focused work, while the renewed increase in the final phase parallels evidence that successful late‐stage therapy sessions typically re‐emphasize patients' strengths and achieved changes to promote self‐responsibility and self‐efficacy (Smith and Grawe 2005).

### 4.1 Limitations and Future Directions

A primary limitation concerns the interpretation of temporal relationships. Although the analyses examined associations between within‐treatment processes (i.e., resource activation) and post‐treatment outcomes (i.e., resource utilization) while adjusting for baseline levels, both resource activation and resource utilization reflect processes unfolding over the course of therapy. Thus, bidirectional or reciprocal relationships between process variables and outcomes cannot be ruled out.

In addition, causal inferences cannot be drawn due to potential confounding factors. Third variables may have influenced both resource activation during therapy and post‐treatment psychosocial resource utilization.

Future studies could investigate within‐person effects in session‐to‐session designs, as these are unaffected by time‐invariant variables and therefore provide stronger – though not definite – evidence for causal relationships (Usami et al. 2019; Wester et al. 2024). For example, Wrede et al. (2024) showed that within‐person resource activation was associated with greater next‐session coping in highly distressed caregivers. However, our study specifically examined whether resource activation during therapy was associated with post‐therapy resource utilization, as theoretically postulated. Although causal inferences remain limited, our findings offer initial evidence consistent with this assumption and underscore the relevance of further process‐outcome analyses to refine our understanding of the role of resource activation. While psychosocial resource utilization represents the theoretically most proximal indicator of successful resource activation, future studies should examine whether changes in resource utilization also translate into improvements in broader clinical outcomes such as caregiver burden or depressive symptoms. Evidence from this trial suggests that increases in psychosocial resource utilization mediate improvements in caregivers' quality of life (Töpfer and Wilz 2021).

A methodological limitation is that resource activation was modeled as a function of session number rather than natural time. This approach aligns with the session‐bound nature of the construct and is supported by evidence that natural‐time modeling does not improve model fit for other process variables such as the working alliance (Wester et al. 2025). However, because session intervals in the intervention systematically varied across the three phases (weekly, biweekly, and monthly), differences in growth patterns may partly reflect session spacing rather than purely therapeutic dynamics. Future studies should therefore examine whether modeling change in natural time yields comparable trajectories and offer complementary insights into how therapeutic processes unfold across varying scheduling formats.

A further limitation concerns the Bern Post Session Report Form 2000, whose items and response scales differ between patient and therapist versions, precluding direct comparisons. This complicates the interpretation of the finding that only caregiver‐rated, but not therapist‐rated, resource activation was associated with post‐therapy psychosocial resource utilization. It also limits comparability with the study by Wrede et al. (2023), which used a different instrument to assess resource activation and found that therapist‐rated, but not caregiver‐rated, resource activation reduced caregiver burden in telephone‐based CBT for distressed family caregivers. Using a measure with corresponding patient and therapist versions, such as the SACiP (Mander et al. 2013), would enable more direct examinations of perspective congruence in future research.

Another limitation is that resource activation was assessed through post‐session self‐report measures. Such retrospective ratings may reflect generalized impressions rather than specific therapeutic behaviors (Hill and Norcross 2023). Future research could complement self‐reports with observational measures, such as coding verbal response modes from session transcripts or recordings. However, such methods may not fully capture patients' internal experiences, which, as previously discussed, are particularly relevant for resource activation.

The interaction between resource activation and other candidate change mechanisms warrants further investigation. In Grawe's (2004) dual therapy model, resource activation represents the process strand aimed at increasing need‐fulfilling experiences, whereas problem actuation reflects the disorder‐specific method strand focused on successful problem processing. Prior research indicates that problem actuation leads to better session outcomes only when supported by high levels of resource activation (Gassmann and Grawe 2006). Recent work further suggests that a slight predominance of resource activation over problem actuation is most predictive of next‐session coping experiences (Wrede et al. 2025), and that resource activation in specific sessions is particularly beneficial for caregivers with higher average levels of problem actuation (Wrede et al. 2024), suggesting that resource activation should complement rather than replace problem actuation. However, little is known about how trajectories of resource activation and problem actuation unfold across different therapy phases, highlighting an important avenue for future research.

Lastly, the significant variance in growth parameters indicates that individual trajectories deviated substantially from the average pattern of change. For caregiver‐rated resource activation, all four growth parameters showed significant variance. For therapist‐rated resource activation, significant variance emerged for the first session and the middle therapy phase. Although baseline resource utilization accounted for some of this interindividual variability, future studies should examine additional predictors of this variability and how patient, therapist, and therapy process characteristics influence the degree of resource activation.

## 5 Conclusion

The findings show that mean resource activation assessed from the caregiver's perspective over the course of telephone‐based CBT for family caregivers of people with dementia was significantly associated with post‐therapy utilization of resources related to well‐being, coping with daily hassles, and social support as a proximal indicator of successful resource activation. Resource activation at the beginning of therapy appeared particularly relevant: caregivers who experienced higher levels of resource activation early in therapy reported more frequent utilization of well‐being and social support resources after therapy. These findings highlight the relevance of resource activation as a candidate general change mechanism in psychotherapy that warrants further investigation and may be integrated as a superordinate intervention heuristic.

## Conflicts of Interest

The authors declare no conflicts of interest.

## Supporting information


**Figure S1:** Johnson‐Neyman plot of the interaction between baseline utilization of coping resources and mean caregiver‐rated resource activation in predicting post‐therapy utilization of coping resources.

Supporting File

## Data Availability

The data that support the findings of this study are available from the corresponding author upon reasonable request.
