# Supplementary material for: From Resource Activation to Utilization: A Process‐Outcome Analysis in Telephone‐Based Cognitive‐Behavioral Therapy for Family Caregivers of People With Dementia
Source: J Clin Psychol. 2026 Jul 15;82(10):1540–52. doi: 10.1002/jclp.70180 (PMC13544484; doi:10.1002/jclp.70180)
Supplement: Supplementary file 1 [file JCLP-82-1540-s001.pdf]

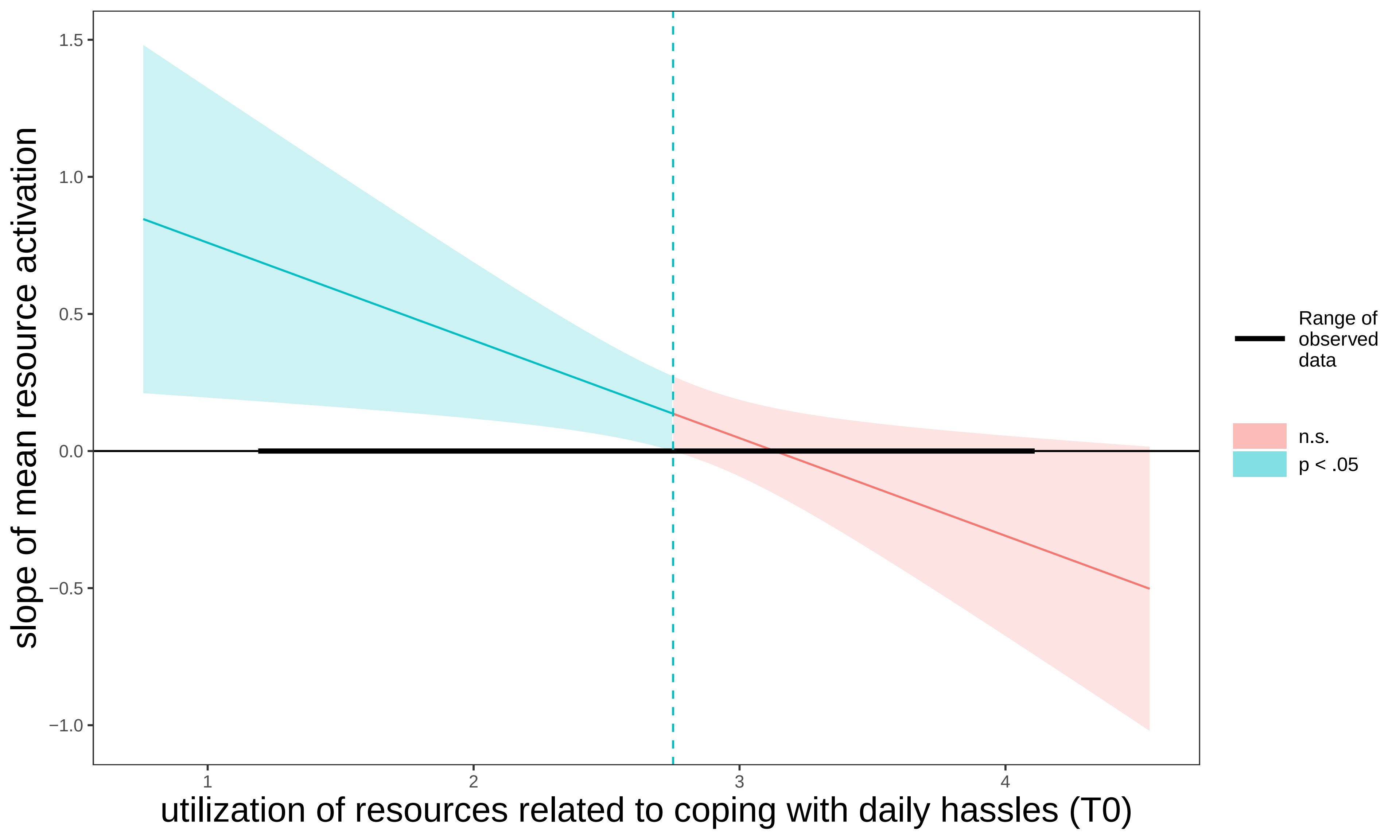

slope of mean resource activation
1.5
1.0
0.5
0.0
−0.5
−1.0
1
2
3
4
utilization of resources related to coping with daily hassles (T0)
Range of observed data
n.s.
p < .05
