# Supplementary material for: From Resource Activation to Utilization: A Process‐Outcome Analysis in Telephone‐Based Cognitive‐Behavioral Therapy for Family Caregivers of People With Dementia
Source: J Clin Psychol. 2026 Jul 15;82(10):1540–52. doi: 10.1002/jclp.70180 (PMC13544484; doi:10.1002/jclp.70180)
Supplement: Supplementary file 2 [file JCLP-82-1540-s002.docx]

```
## Analyses for
## Resource activation as a change mechanism in
## telephone-based cognitive-behavioral therapy
## for family caregivers of people with dementia:
## Findings from process-outcome analyses
## Nils F. Töpfer, Robin A. Wester, & Gabriele Wilz
## script: R. A. Wester


library(haven)
library(tidyverse)
library(magrittr)
library(lm.beta)
library(interactions)
library(lavaan)

#-----------------------------##
## mean resource activation ####
#-----------------------------##


#-------------------------#
## caregiver perspective ##

## social support
ang_regr_RES_un0 <- lm(RES_unT1 ~ RES_unT0, sub.proz.out)
ang_regr_RES_un1 <- lm(RES_unT1 ~ PSTB_RA_mean + RES_unT0, sub.proz.out)
ang_regr_RES_un2 <- lm(RES_unT1 ~ PSTB_RA_mean * RES_unT0, sub.proz.out)
anova(ang_regr_RES_un0, ang_regr_RES_un1, ang_regr_RES_un2)
summary(lm.beta(ang_regr_RES_un1))
round(coef(summary(lm.beta(ang_regr_RES_un1))),2)

# well-being
ang_regr_RES_wohlb0 <- lm(RES_wohlbefT1 ~ RES_wohlbefT0, sub.proz.out)
ang_regr_RES_wohlb1 <- lm(RES_wohlbefT1 ~ PSTB_RA_mean + RES_wohlbefT0, sub.proz.out)
ang_regr_RES_wohlb2 <- lm(RES_wohlbefT1 ~ PSTB_RA_mean * RES_wohlbefT0, sub.proz.out)
anova(ang_regr_RES_wohlb0, ang_regr_RES_wohlb1, ang_regr_RES_wohlb2)
summary(lm.beta(ang_regr_RES_wohlb1))
round(summary(lm.beta(ang_regr_RES_wohlb1))$r.squared,2)
round(coef(summary(lm.beta(ang_regr_RES_wohlb1))),2)

## coping with daily hassles
ang_regr_RES_bew0 <- lm(RES_bewstressT1 ~ RES_bewstressT0, sub.proz.out)
ang_regr_RES_bew1 <- lm(RES_bewstressT1 ~ PSTB_RA_mean + RES_bewstressT0, sub.proz.out)
ang_regr_RES_bew2 <- lm(RES_bewstressT1 ~ PSTB_RA_mean * RES_bewstressT0, sub.proz.out)
anova(ang_regr_RES_bew0, ang_regr_RES_bew1, ang_regr_RES_bew2)
summary(lm.beta(ang_regr_RES_bew2))
round(coef(summary(lm.beta(ang_regr_RES_bew2))),2)
round(summary(lm.beta(ang_regr_RES_bew2))$r.squared,2)
jnplot <- johnson_neyman(ang_regr_RES_bew2, pred = PSTB_RA_mean, modx = RES_bewstressT0, title = NULL)
jnplot$plot +
  labs(x = "utilization of resources related to coping with daily hassles (T0)", y = "slope of mean resource activation") +
  theme(axis.title.x = element_text(size = 18),
        axis.title.y = element_text(size = 18))

ggsave("Figure_S1.pdf", width = 10, height = 6, dpi = 1200, device = "pdf")

# interact_plot(ang_regr_RES_bew2, pred = PSTB_RA_mean, modx = RES_bewstressT0, plot.points = TRUE)
sim_slopes(ang_regr_RES_bew2, pred = PSTB_RA_mean, modx = RES_bewstressT0, modx.values = c(1.2, 2.75))


#----------------------#
## therapist perspective
## social support
the_regr_RES_un0 <- lm(RES_unT1 ~ RES_unT0, sub.proz.out)
the_regr_RES_un1 <- lm(RES_unT1 ~ TSTB_RA_mean + RES_unT0, sub.proz.out)
the_regr_RES_un2 <- lm(RES_unT1 ~ TSTB_RA_mean * RES_unT0, sub.proz.out)
anova(the_regr_RES_un0, the_regr_RES_un1, the_regr_RES_un2)
summary(lm.beta(the_regr_RES_un1))
round(summary(lm.beta(the_regr_RES_un1))$r.squared,2)
round(coef(summary(lm.beta(the_regr_RES_un1))),2)

# well-being
the_regr_RES_wohlb0 <- lm(RES_wohlbefT1 ~ RES_wohlbefT0, sub.proz.out)
the_regr_RES_wohlb1 <- lm(RES_wohlbefT1 ~ TSTB_RA_mean + RES_wohlbefT0, sub.proz.out)
the_regr_RES_wohlb2 <- lm(RES_wohlbefT1 ~ TSTB_RA_mean * RES_wohlbefT0, sub.proz.out)
anova(the_regr_RES_wohlb0, the_regr_RES_wohlb1, the_regr_RES_wohlb2)
summary(lm.beta(the_regr_RES_wohlb1))
round(summary(lm.beta(the_regr_RES_wohlb1))$r.squared,2)
round(coef(summary(lm.beta(the_regr_RES_wohlb1))),2)

## coping with daily hassles
the_regr_RES_bew0 <- lm(RES_bewstressT1 ~ RES_bewstressT0, sub.proz.out)
the_regr_RES_bew1 <- lm(RES_bewstressT1 ~ TSTB_RA_mean + RES_bewstressT0, sub.proz.out)
the_regr_RES_bew2 <- lm(RES_bewstressT1 ~ TSTB_RA_mean * RES_bewstressT0, sub.proz.out)
anova(the_regr_RES_bew0, the_regr_RES_bew1, the_regr_RES_bew2)
summary(lm.beta(the_regr_RES_bew1))
round(summary(lm.beta(the_regr_RES_bew1))$r.squared,2)
round(coef(summary(lm.beta(the_regr_RES_bew1))),2)


#---------------------------------------#
## trajectory of resource activation ####
#---------------------------------------#

# caregiver perspective: unconditional model
caregiv_unc_mod <- '
# intercept
eta_0 =~ 1*PSTB_RA_01 + 1*PSTB_RA_02 + 1*PSTB_RA_03 + 1*PSTB_RA_04 + 1*PSTB_RA_05 + 1*PSTB_RA_06 + 1*PSTB_RA_07 + 1*PSTB_RA_08 + 1*PSTB_RA_09 + 1*PSTB_RA_10 + 1*PSTB_RA_11 + 1*PSTB_RA_12
# slope 1
eta_1 =~ 0*PSTB_RA_01 + 1*PSTB_RA_02 + 2*PSTB_RA_03 + 3*PSTB_RA_04 + 3*PSTB_RA_05 + 3*PSTB_RA_06 + 3*PSTB_RA_07 + 3*PSTB_RA_08 + 3*PSTB_RA_09 + 3*PSTB_RA_10 + 3*PSTB_RA_11 + 3*PSTB_RA_12
# slope 2
eta_2 =~ 0*PSTB_RA_01 + 0*PSTB_RA_02 + 0*PSTB_RA_03 + 0*PSTB_RA_04 + 1*PSTB_RA_05 + 2*PSTB_RA_06 + 3*PSTB_RA_07 + 4*PSTB_RA_08 + 5*PSTB_RA_09 + 6*PSTB_RA_10 + 6*PSTB_RA_11 + 6*PSTB_RA_12
# slope 3
eta_3 =~ 0*PSTB_RA_01 + 0*PSTB_RA_02 + 0*PSTB_RA_03 + 0*PSTB_RA_04 + 0*PSTB_RA_05 + 0*PSTB_RA_06 + 0*PSTB_RA_07 + 0*PSTB_RA_08 + 0*PSTB_RA_09 + 0*PSTB_RA_10 + 1*PSTB_RA_11 + 2*PSTB_RA_12
'
fit.caregiv_unc_mod <- growth(caregiv_unc_mod, sub.proz.out, estimator = "MLR", missing = "fiml")
summary(fit.caregiv_unc_mod, fit.measures = T)
round(lavInspect(fit.caregiv_unc_mod, what = "mean.lv"), 3)
round(lavInspect(fit.caregiv_unc_mod, what = "cov.lv"), 3)

# caregiver predictive model
caregiv_pred_mod <- '
# intercept
eta_0 =~ 1*PSTB_RA_01 + 1*PSTB_RA_02 + 1*PSTB_RA_03 + 1*PSTB_RA_04 + 1*PSTB_RA_05 + 1*PSTB_RA_06 + 1*PSTB_RA_07 + 1*PSTB_RA_08 + 1*PSTB_RA_09 + 1*PSTB_RA_10 + 1*PSTB_RA_11 + 1*PSTB_RA_12
# slope 1
eta_1 =~ 0*PSTB_RA_01 + 1*PSTB_RA_02 + 2*PSTB_RA_03 + 3*PSTB_RA_04 + 3*PSTB_RA_05 + 3*PSTB_RA_06 + 3*PSTB_RA_07 + 3*PSTB_RA_08 + 3*PSTB_RA_09 + 3*PSTB_RA_10 + 3*PSTB_RA_11 + 3*PSTB_RA_12
# slope 2
eta_2 =~ 0*PSTB_RA_01 + 0*PSTB_RA_02 + 0*PSTB_RA_03 + 0*PSTB_RA_04 + 1*PSTB_RA_05 + 2*PSTB_RA_06 + 3*PSTB_RA_07 + 4*PSTB_RA_08 + 5*PSTB_RA_09 + 6*PSTB_RA_10 + 6*PSTB_RA_11 + 6*PSTB_RA_12
# slope 3
eta_3 =~ 0*PSTB_RA_01 + 0*PSTB_RA_02 + 0*PSTB_RA_03 + 0*PSTB_RA_04 + 0*PSTB_RA_05 + 0*PSTB_RA_06 + 0*PSTB_RA_07 + 0*PSTB_RA_08 + 0*PSTB_RA_09 + 0*PSTB_RA_10 + 1*PSTB_RA_11 + 2*PSTB_RA_12
# regressions
eta_0 ~ RES_bewstressT0 + RES_unT0 + RES_wohlbefT0
eta_1 ~ RES_bewstressT0 + RES_unT0 + RES_wohlbefT0
eta_2 ~ RES_bewstressT0 + RES_unT0 + RES_wohlbefT0
eta_3 ~ RES_bewstressT0 + RES_unT0 + RES_wohlbefT0
RES_bewstressT1 ~ eta_0 + eta_1 + eta_2 + eta_3 + RES_bewstressT0 + RES_wohlbefT0 + RES_unT0
RES_unT1 ~ eta_0 + eta_1 + eta_2 + eta_3 + RES_bewstressT0 + RES_wohlbefT0 + RES_unT0
RES_wohlbefT1 ~ eta_0 + eta_1 + eta_2 + eta_3 + RES_bewstressT0 + RES_wohlbefT0 + RES_unT0
# intercepts
RES_bewstressT1 ~ 1
RES_unT1 ~ 1
RES_wohlbefT1 ~ 1
'
fit.caregiv_pred_mod <- growth(caregiv_pred_mod, sub.proz.out, estimator = "MLR", missing = "fiml")
summary(fit.caregiv_pred_mod, fit.measures = T)
round(lavInspect(fit.caregiv_pred_mod, what = "coef")$beta,2)
# lavInspect(fit.ang_res_piecewise, what = "mean.lv")
# lavInspect(fit.ang_res_piecewise, what = "est")
# fitted(fit.ang_res_piecewise)
# parameterEstimates(fit.ang_res_piecewise)


# therapist perspective: unconditional model
therap_unc_mod <- '
# intercept
eta_0 =~ 1*TSTB_RA_01 + 1*TSTB_RA_02 + 1*TSTB_RA_03 + 1*TSTB_RA_04 + 1*TSTB_RA_05 + 1*TSTB_RA_06 + 1*TSTB_RA_07 + 1*TSTB_RA_08 + 1*TSTB_RA_09 + 1*TSTB_RA_10 + 1*TSTB_RA_11 + 1*TSTB_RA_12
# slope 1
eta_1 =~ 0*TSTB_RA_01 + 1*TSTB_RA_02 + 2*TSTB_RA_03 + 3*TSTB_RA_04 + 3*TSTB_RA_05 + 3*TSTB_RA_06 + 3*TSTB_RA_07 + 3*TSTB_RA_08 + 3*TSTB_RA_09 + 3*TSTB_RA_10 + 3*TSTB_RA_11 + 3*TSTB_RA_12
# slope 2
eta_2 =~ 0*TSTB_RA_01 + 0*TSTB_RA_02 + 0*TSTB_RA_03 + 0*TSTB_RA_04 + 1*TSTB_RA_05 + 2*TSTB_RA_06 + 3*TSTB_RA_07 + 4*TSTB_RA_08 + 5*TSTB_RA_09 + 6*TSTB_RA_10 + 6*TSTB_RA_11 + 6*TSTB_RA_12
# slope 3
eta_3 =~ 0*TSTB_RA_01 + 0*TSTB_RA_02 + 0*TSTB_RA_03 + 0*TSTB_RA_04 + 0*TSTB_RA_05 + 0*TSTB_RA_06 + 0*TSTB_RA_07 + 0*TSTB_RA_08 + 0*TSTB_RA_09 + 0*TSTB_RA_10 + 1*TSTB_RA_11 + 2*TSTB_RA_12
'
fit.therap_unc_mod <- growth(therap_unc_mod, sub.proz.out, estimator = "MLR", missing = "fiml")
summary(fit.therap_unc_mod, fit.measures = T)
round(lavInspect(fit.therap_unc_mod, what = "mean.lv"), 2)
round(lavInspect(fit.therap_unc_mod, what = "cov.lv"), 3)

therap_pred_mod <- '
# intercept
eta_0 =~ 1*TSTB_RA_01 + 1*TSTB_RA_02 + 1*TSTB_RA_03 + 1*TSTB_RA_04 + 1*TSTB_RA_05 + 1*TSTB_RA_06 + 1*TSTB_RA_07 + 1*TSTB_RA_08 + 1*TSTB_RA_09 + 1*TSTB_RA_10 + 1*TSTB_RA_11 + 1*TSTB_RA_12
# slope 1
eta_1 =~ 0*TSTB_RA_01 + 1*TSTB_RA_02 + 2*TSTB_RA_03 + 3*TSTB_RA_04 + 3*TSTB_RA_05 + 3*TSTB_RA_06 + 3*TSTB_RA_07 + 3*TSTB_RA_08 + 3*TSTB_RA_09 + 3*TSTB_RA_10 + 3*TSTB_RA_11 + 3*TSTB_RA_12
# slope 2
eta_2 =~ 0*TSTB_RA_01 + 0*TSTB_RA_02 + 0*TSTB_RA_03 + 0*TSTB_RA_04 + 1*TSTB_RA_05 + 2*TSTB_RA_06 + 3*TSTB_RA_07 + 4*TSTB_RA_08 + 5*TSTB_RA_09 + 6*TSTB_RA_10 + 6*TSTB_RA_11 + 6*TSTB_RA_12
# slope 3
eta_3 =~ 0*TSTB_RA_01 + 0*TSTB_RA_02 + 0*TSTB_RA_03 + 0*TSTB_RA_04 + 0*TSTB_RA_05 + 0*TSTB_RA_06 + 0*TSTB_RA_07 + 0*TSTB_RA_08 + 0*TSTB_RA_09 + 0*TSTB_RA_10 + 1*TSTB_RA_11 + 2*TSTB_RA_12
# regressions
eta_0 ~ RES_bewstressT0 + RES_unT0 + RES_wohlbefT0
eta_1 ~ RES_bewstressT0 + RES_unT0 + RES_wohlbefT0
eta_2 ~ RES_bewstressT0 + RES_unT0 + RES_wohlbefT0
eta_3 ~ RES_bewstressT0 + RES_unT0 + RES_wohlbefT0
RES_bewstressT1 ~ eta_0 + eta_1 + eta_2 + eta_3 + RES_bewstressT0 + RES_wohlbefT0 + RES_unT0
RES_unT1 ~ eta_0 + eta_1 + eta_2 + eta_3 + RES_bewstressT0 + RES_wohlbefT0 + RES_unT0
RES_wohlbefT1 ~ eta_0 + eta_1 + eta_2 + eta_3 + RES_bewstressT0 + RES_wohlbefT0 + RES_unT0
# intercepts
RES_bewstressT1 ~ 1
RES_unT1 ~ 1
RES_wohlbefT1 ~ 1
'
fit.therap_pred_mod <- growth(therap_pred_mod, sub.proz.out, estimator = "MLR", missing = "fiml")
summary(fit.therap_pred_mod, fit.measures = T)
round(lavInspect(fit.therap_pred_mod, what = "coef")$beta,2)
```
